# The Effect of Learning Burnout on Sleep Quality in Primary School Students: The Mediating Role of Mental Health

**DOI:** 10.3390/healthcare10102076

**Published:** 2022-10-19

**Authors:** Lulu Qin, Si Chen, Bangan Luo, Yiwei Chen

**Affiliations:** 1 Department of Social Medicine and Health Management, School of Medicine, Hunan Normal University, Changsha 410013, China; 2 Department of Mental Health, Brain Hospital of Hunan Province (The Second People’s Hospital of Hunan Province), Changsha 410007, China; 3 Department of Neurology, Xiangya Third Hospital, Central South University, Changsha 410017, China

**Keywords:** primary students, sleep quality, learning burnout, mental health

## Abstract

Due to the growth of research on sleep, mental health, and learning burnout on healthy growth and its related public health significance of adolescents, this study aimed to provide a deeper understanding of the effect of mental health and learning burnout on sleep among primary school students. The sleep quality (subjective sleep quality, sleep time, sleep latency, sleep duration, sleep efficiency, sleep disturbance, and daytime dysfunction), mental health, and learning burnout (exhaustion, learning cynicism, and reduced efficacy) of 900 students of grades 3–6 in primary schools were assessed in 2020. The PSQI scores of participants were 4.19 ± 2.545, of which a number of 322 (39.03%) students had sleep disturbance (PSQI scores ≧ 5). Binary logistic regression analysis showed that screen time (*OR* = 1.518, 95% *CI*: 1.164–1.980), ranking status (*OR* = 0.659, 95% *CI*: 0.480–0.907), learning burnout (*OR* = 1.088, 95% *CI*: 1.067–1.108), and mental health (*OR* = 4.672, 95% *CI*: 1.954–11.173) were the influencing factors for sleep quality of grade 3–6 students. According to the mediation effect analyses, mental health played a mediating effect (58.73% of the total effect) on the relationship between learning burnout and sleep quality. In conclusion, primary school students in Hunan of China have prominent sleep problems, and the daytime dysfunction caused by sleep problems is the most serious. Learning burnout positively predicted poorer sleep quality, and mental health played a mediating effect on the relationship between learning burnout and sleep quality.

## 1. Introduction

Sleep is closely related to the healthy growth of adolescents and has extensive public health significance. The American Academy of Sleep Medicine proposes that school-age children need to ensure 9–12 h of sleep to maintain their physical and mental health [1] Good sleep quality is conducive to the healthy growth, study, and life of adolescents. However, many studies have reported that children have many sleep-related problems. Hawkins et al. reported that sleep deprivation occurred as early as age 6, and that sleep deprivation became more common with increased age and showed a social pattern [2]. Research by Norell-Clarke et al. found that fewer children and adolescents in Sweden got enough sleep over time, which may have an impact on their mental health and cognitive abilities [3].

Mental health is a state of well-being, in which every individual who realizes his or her own potential can cope with the normal stresses of life, can work productively and fruitfully, and is able to make a contribution to her or his community [4]. In the theory of psychological development, psychologist, Erickson, proposed that childhood (6–11 years old) is the basic stage of individual physical and mental health development and the key to ensuring adult mental health [5]. However, studies at home and abroad have shown that the incidence of children’s mental health problems has become increasingly serious [6]. Compared with a decade ago, British teenagers had higher rates of depression and self-harm; parents reported higher levels of emotional difficulties, conduct problems, hyperactivity and peer problems, and less sleep [7].

Burnout is defined as a syndrome composed of three factors: Depersonalization, emotional exhaustion and reduced personal achievement [8]. With the gradual deepening of the research on burnout, learning burnout is summarized as a negative emotion caused by the long-term learning pressure and workload of students, leading to their loss of interest in learning activities [9]. Current studies show that learning burnout is common among college students, which can bring negative psychological and behavioral consequences, including anxiety, depression, and so on [10]. According to a study in Nanjing of China, the rate of learning burnout among college students reached a staggering 90.3% [11]. Primary school is the initial stage of learning, and a good learning attitude is conducive to improving the efficiency and quality of learning. However, current research on learning burnout mainly focuses on college students and professional learners, while the research on learning burnout of primary school students is lack obviously.

Many previous studies have shown that learning burnout is associated with sleep quality [7,11,12]. Meanwhile, it is known that learning burnout leads to many mental health problems, such as anxiety and depression [13]. In addition, mental health was related to one’s sleep quality, for example, according to a Canadian study, sleep quality was correlated with screening measures for post-traumatic stress disorder (PTSD), depression, anxiety, social anxiety disorder, panic disorder, and alcohol-use disorder for all Public Safety Personnel categories [14]. A prospective study has shown that burnout can have an impact on mental health, with adverse symptoms such as insomnia, depressive symptoms, use of psychotropic and antidepressant medications, hospitalization for mental disorders, and poor mental health [15]. In China, previous studies have shown that the learning burnout of primary school students was at a medium level [16,17], and even some studies have reported high levels in China’s primary students [18], which should be concerning as a serious problem. Primary school is only the initial stage of learning life; students will face heavier learning tasks and greater pressure as they grow up. However, in past studies, researchers used learning burnout, mental health, and sleep quality as outcome variables to explore their relationships with other factors. However, no study focuses on the direct connection between them and discusses the effect paths between them, such as mediating effects. Thus, early prevention of learning burnout in primary school students should be paid attention to, and good sleep quality and mental health may be conducive to reducing learning burnout and improving learning efficiency.

Due to the deficiency and importance of the study on the effect paths among the three variables (learning burnout, mental health, and sleep quality) among primary students, it is necessary to point out how the above factors affect each other. As far as we know, this is the first study to explore the effect on primary school students’ sleep quality from the perspective of mental health and learning burnout. In this study, we took sleep quality as the dependent variable and planned to explore the sleep quality of primary school students from the perspective of mental health and learning burnout, as well as the mechanism and way that mental health and learning burnout affect sleep quality. Research hypothesis: H1: Learning burnout can positively predict the poor sleep quality of primary school students; H2: Mental health plays a mediating role in the relationship between sleep quality and learning burnout.

## 2. Methods

### 2.1. Participants and Procedures

This study was a cross-sectional survey conducted in the Hunan province of China. In consideration of reading and writing skills, primary students of grades 3–6 were selected as the survey objects by using the cluster sampling method. Data were collected through self-filling questionnaires, which were distributed and collected by researchers in the class in 7 primary schools. A number of 900 questionnaires were sent out in this survey, and a total of 825 valid questionnaires were collected with an efficiency rate of 91.67% (825/900).

### 2.2. Measuring Instruments

#### 2.2.1. Socio-Demographic Information

Gender (boy or girl), age, grade, height (cm), weight (cm), learning performance (highly ranked: students’ performance measured by teacher ranked in the top one-third of their class, middle-ranked: students’ performance measured by teacher ranked in the middle one-third of their class, lower ranked: students’ performance measured by teacher ranked in the bottom one-third of their class), screen time in a day (hours), myopia (yes or no), having brothers and sisters (yes or no), primary caregiver (parents or others), having the habit of an afternoon nap in the past month (yes or no). BMI was computed with the following formula: BMI = kg/m^2^. Participants were defined as being lean, normal, overweight, or obese according to the Chinese standards of children [19]. Screen time was defined as time spent on screen devices, including time spent watching TV, using computers, using mobile phones, and using video games and e-readers [20].

#### 2.2.2. Sleep Quality

The Chinese version of the Pittsburgh Sleep Quality Index (PSQI) was used to measure the sleep quality of primary students [21], which was used to evaluate the sleep quality of individuals in the past 30 days. PSQI is composed of seven factors: subjective sleep quality, sleep time, sleep latency, sleep duration, sleep efficiency, sleep disturbance, and daytime dysfunction. Each factor was scored by a four-level scale of 0–3, with a total score range of 0–21. PSQI ≥ 5 indicates significant sleep disturbance, and the higher the score, the worse the sleep quality. The PSQI was verified by Buysse et al. and showed good reliability; Cronbach’s α was 0.83. The Chinese version of PSQI has been tested and proved to be an effective clinical tool with good reliability, and has proved to be suitable for investigation among children [22].

#### 2.2.3. Mental Health

The Kessler Psychological Distress Scale (K6) [23] was used to measure the mental health of primary school students. This scale contains 6 items, and participants are required to self-evaluate according to the frequency of distress symptoms in the recent month. A five-level score of 0–4 is adopted, with a total score ≤12 indicating a low risk of psychological disorders and ≥13 indicating a high risk of psychological disorders. In recent years, K6 has been proven to be a good response to adolescent mental health in many studies, and its simplicity and strong predictive ability for mental illness have confirmed its usefulness in clinical and community settings [24]. In addition, Cronbach’s α coefficient of the K6 was 0.89.

#### 2.2.4. Learning Burnout

Adolescent Student Burnout Inventory (ASBI) [25] was used to evaluate the learning burnout of primary school students. The scale is a self-rating scale with three dimensions: exhaustion, learning cynicism, and reduced efficacy. There are a total of 16 items on the scale, and a five-point scoring method is adopted: “very consistent”—5 points, “somewhat consistent”—4 points, “not quite consistent”—3 points, “not quite consistent”—2 points, and “Very inconsistent”—1 point and some items in the scale need reverse scoring. ASBI has good reliability, validity, and internal consistency in primary school students [26]. Additionally, Cronbach’s α coefficient of ASBI was 0.91.

### 2.3. Statistical Analysis

Epidata 3.1 software was used for data entry, SPSS 23.0 was used for data statistical analysis. Pearson correlation analysis was used to explore the relationship between learning burnout, sleep quality, and mental health. Binary logistic regression analysis with sleep quality as an independent variable was performed to explore the influencing factors of sleep quality of grades 3–6 primary students. Drawing using Amos 21.0 software [27].

The common method deviation test was performed using Harman single-factor test, the exploratory factor analysis was conducted on all items of primary school students learning burnout, mental health, and sleep quality. Meanwhile, model 4 in the SPSS process compiled by Hayes (2012) [28] (model 4 is a simple mediation model) was used to test the mediation effect of mental health in the relationship between learning burnout and sleep quality under the control of age, grade, ranking status, screen time, and whether they had the habit of napping in the past month (the level α = 0.05).

## 3. Results

### 3.1. Socio-Demographic Information of Participants

Among the 825 primary students, 400 (48.5%) were boys and 425 (51.5%) were girls. A total of 558 (67.6%) students reported less than one hour of screen time per day, 216 (26.2%) students reported 1–2 h of screen time per day, and 51 (6.2%) students reported more than 2 h of screen time per day. More information about the participants is shown in Table 1.

### 3.2. Sleep Quality of the Primary Students in Grades 3–6 and Its Influencing Factors

The PSQI scores of 825 students of grades 3–6 were (4.19 ± 2.545), of which a number of 322 (39.03%) students were with sleep disturbance (PSQI scores ≧ 5). Among the seven dimensions of the PSQI, the highest score was daytime dysfunction and the lowest was hypnotic medication use (Table 2 and Tables S1–S3).

### 3.3. Exploring the Influencing Factors of Sleep Quality of Grades 3–6 Students

Binary logistic regression analysis showed that screen time (*OR* = 1.518, 95%*CI*: 1.164–1.980), ranking status (*OR* = 0.659, 95%*CI*: 0.480–0.907), learning burnout (*OR* = 1.088, 95%*CI*: 1.067–1.108), and mental health (*OR* = 4.672, 95%*CI*: 1.954–11.173) were the influencing factors for sleep quality of grade 3–6 students. The specific results are shown in Table 3 below.

### 3.4. Mental Health and Learning Burnout and Its Correlations with Sleep Quality of Grades 3–6 Students

The average score of mental health of primary school students in grades 3–6 was 4.46 ± 4.404, and 45 students (5.5%) scored 13 or above, indicating that the participants had a high risk of psychological disorders. (Supplementary Table S4).

The average score of learning burnout of the grades 3–6 students was 36.23 ± 10.383. Among the three dimensions of learning burnout, the reduced efficacy dimension scored the highest (17.33 ± 4.726), and the learning cynicism scored the lowest (8.71 ± 4.213) (Supplementary Table S5).

Correlation analysis showed that sleep quality significant positive correlated with mental health (r = 0.551, *p* < 0.05) and learning burnout (r = 0.505, *p* < 0.05). There was a significant positive correlation between mental health and learning burnout (r = 0.542, *p* < 0.05) (Supplementary Table S6).

### 3.5. Mediating Effect of Mental Health on the Relationship between Learning Burnout and Sleep Quality

**Figure 1:**
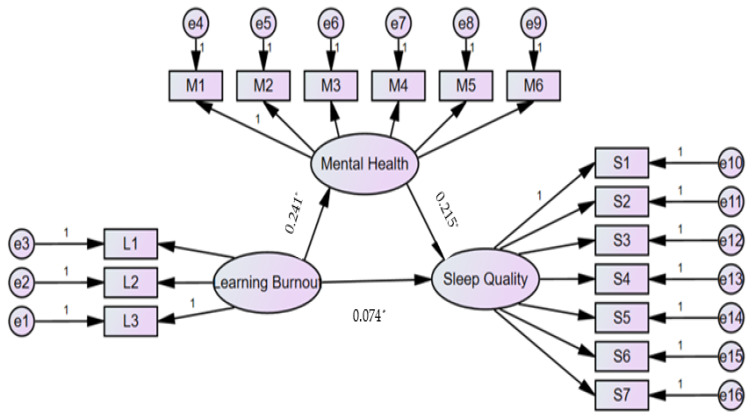
Mediating role of mental health. Note: L1, L2, and L3 are the three dimensions of learning burnout, respectively; M1–M6 are the 6 questions of K6, respectively; S1–S7 are seven dimensions of sleep quality, respectively; e1–e16 are the residual error to each variable; The number 1 means that the path coefficient (i.e., the influence coefficient) has a fixed value of 1; The asterisk (*) means that the path coefficient is statistically significant.

Results from the mediating effect analysis showed that learning burnout had a significant predictive effect on sleep quality (*β* = 0.126, *t* = 15.621, *p* < 0.001), and the direct predictive effect of learning burnout on sleep quality was still significant (*β* = 0.074, *t* = 8.415, *p* < 0.001). Learning burnout has a significant positive predictive effect on mental health (*β* = 0.241, *t* = 17.587, *p* < 0.001); the positive predictive effect of mental health on sleep quality was also significant (*β* = 0.215, *t* = 11.203, *p* < 0.001). In addition, learning burnout can not only directly predict sleep quality, but also predict sleep quality through the intermediary effect of mental health. Mental health played a mediating effect (58.73% of the total effect) on the relationship between learning burnout and sleep quality (Table 4 and Table S6 and Figure 1).

## 4. Discussion

### 4.1. Sleep Quality of the Primary Students in Grades 3–6

This study showed that the PSQI score of pupils in grades 3–6 of China was 4.19 ± 2.545, which was significantly lower than the critical value of 5 (*t* = −9.136, *p* = 0.000) and the global average score of PSQI (*t* = −4.961, *p* = 0.000). Compared with previous studies of Chongqing City of China in 2011 (PSQI score: 5.51 ± 2.57), Hunan primary school students have better sleep quality than them [29]. In 2021, a Portuguese study showed that the PSQI reported by children aged 6–10 was 4.05 ± 2.803, slightly lower than the score of this study [30].

The prevalence of sleep disorders in primary school students of grades 3–6 in Hunan Province was 39%, which was similar to developing countries. The prevalence of sleep disorders in children is estimated to be 20–50% and varies in different areas of the world, such as nearly 40% in Japan in 2013 [31] and 43.1% in Spain in 2016 [32]. According to the regulations of the Ministry of Education in China, the sleep time of primary school students should be more than 10 h [33]. Unfortunately, only 166 (20.12%) primary school students in this survey slept for 10 h and above, suggesting that the sleep problems of primary school students in Hunan were serious.

Among the seven dimensions of PSQI, the score of the daytime dysfunction dimension was the highest, suggesting that daytime dysfunction caused by sleep problems was more serious than others among the participants. The score of the hypnotic medication-use dimension was the lowest, which may be related to the careful thinking of parents. Parents always prefer to be very cautious when using drugs for children. According to the score of the sleep efficiency dimension, it was shown that the sleep efficiency of primary school students is relatively good in this study.

Logistic results showed that the sleep quality of primary school students was influenced by their ranking status, screen time, mental health, and learning burnout. Results showed that children’s sleep quality was significantly correlated with their ranking status in the class. The lower the performance ranking, the worse the sleep quality. Firstly, poor sleep quality is closely related to the adverse effects on the cognitive process of the prefrontal cortex, which may have a negative impact on working memory and executive function [34]. Secondly, poor sleep quality will reduce daytime alertness, which, in turn, may decrease the attention effect and lead to impaired academic performance [35]. In addition, students at the last ranking will encounter greater learning pressure, which may affect the quality of sleep [1,2,3,4]. Screen time was positively correlated with the sleep quality score of primary school students, this may be due to the screen light stimulating the brain and inhibiting melatonin production, resulting in an increase in sleep latency [36].

### 4.2. The Mediating Role of Mental Health on the Influence of Learning Burnout on Sleep Quality of Primary School Students

Our study showed that learning burnout had a significant impact on sleep among primary school students. There are two main pathways to this effect: (1) one is that learning burnout directly affects sleep quality; (2) the other is the effect of learning burnout on sleep quality through the mediating effect of mental health, which was the first time that it was demonstrated that mental health played a mediating effect on the relationship between learning burnout and sleep quality.

As for the first hypothesis of this study: learning burnout can positively predict the poor sleep quality of primary school students, our results show that this hypothesis is valid. There was a moderately positive correlation between learning burnout and sleep quality score (r = 0.505, *p* < 0.05), that is, the higher the learning burnout degree, the worse the sleep quality. This is consistent with previous studies. A Finnish study on middle school students showed that burnout was related to sleep disorders and poor sleep quality [37]. Wolf and Rosenstock reported that pathological sleepiness was significantly associated with a higher incidence of burnout. Sleep deprivation is associated with significantly reduced occupational efficacy and higher fatigue scores [38]. Scholars in the United States found that learning burnout was related to the global Pittsburgh sleep quality total score and its seven subscales [39]. Thus, students with a low level of learning burnout have a strong sense of autonomy and competence. When encountering difficulties, they will actively try more solutions to improve learning efficiency and reduce the pressure brought by learning, which is conducive to developing good sleep.

In our second hypothesis, mental health plays a mediating role in the relationship between learning burnout and sleep quality. The results of the study show that this hypothesis is tenable. Mental health has a mediating effect between learning burnout and sleep quality, and mental health plays a “bridge” role, that is, learning burnout not only directly affects sleep quality, but also indirectly affects sleep quality through mental health. These findings are consistent with other studies [40]. On one hand, learning burnout will have an impact on mental health, and a high degree of burnout will bring many negative effects on mental health. Some Finnish scholars found that burnout is related to dropping out of school, dysfunction caused by mental health problems, and at least one mental illness at present [41]. The effect of learning burnout on mental health can be explained by the JDR model (Job Demands–Resources model) [42]. The JDR model proposed that the matching degree of adolescents’ needs and resources in the school environment affects their school-related burnout. When school resources cannot meet their needs, adolescents may feel high academic pressure. Persistent high academic pressure can lead to physical and mental exhaustion, a lack of personal achievement in learning, and a sense of alienation in their relationship with teachers [40]. In addition, under high study pressure, teenagers tend to produce a series of emotion-related reactions, such as depression. All this evidence suggests that learning burnout can have a negative impact on mental health. On the other hand, the effect of mental health on sleep has been demonstrated in many studies, and a review by Lam [43] literature points to a significant and possible causal relationship between early childhood sleep disorders and the development of mental health problems such as anxiety, depression, and ADHD (Attention Deficit Hyperactivity Disorder) in adolescence. A study of sleep among primary school students in Iran also showed a significant correlation between sleep habits and mental health [44]. Therefore, it can be inferred that learning burnout can affect the mental health of primary school students through learning pressure factors, and the mental health of primary school students will further affect their sleep quality, so the mediating effect of mental health between the two is established.

In view of the results of our study, we intend to put forward some suggestions for education researchers and parents. Firstly, we need to pay enough attention to children’s learning burnout, mental health, and sleep quality to help children grow up healthily. Secondly, teachers and parents should look at the role of study pressure dialectically; moderate study pressure may promote learning, but excessive study pressure may bring many negative effects [45,46]. It is necessary to think about how to maintain moderate study pressure to promote students’ learning. In addition, considering the effective ways of learning burnout, mental health, and sleep quality of this study, some intervention strategies should be working together to provide a better scientific basis for the healthy growth of children and adolescents.

However, there are some limitations to this study and our results should be interpreted with caution. Firstly, we only measure subjective sleep quality. The assessment of objective sleep quality was not conducted in this study. In future studies, the objective sleep quality of children reported could be considered. In addition, due to the cross-sectional design, so the causation cannot be inferred, and future researchers can further verify the relationship using longitudinal methods and/or randomized control designs.

## 5. Conclusions

Primary school students in grades 3–6 have more prominent sleep problems, and the daytime dysfunction caused by sleep problems is the most serious in Hunan of China. Learning burnout positively predicted poorer sleep quality and mental health played a mediating effect on the relationship between learning burnout and sleep quality.

## Figures and Tables

**Table 1:** Socio-demographic information of participants (*n* = 825).

Variables		Number	Percentage (%)
Gender	Boy	400	48.5
	Girl	425	51.5
Age (years)	≤9	219	26.5
	10	237	28.7
	11	164	19.9
	≥12	205	24.8
Grade	The 3 grade	225	27.3
	The 4 grade	213	25.8
	The 5 grade	156	18.9
	The 6 grade	231	28.0
BMI (kg/m^2^)	Lean	118	14.3
	Normal	573	69.5
	Overweight	134	16.2
Myopia	Yes	463	56.1
	No	362	43.9
Ranking status in class	The first one-third ranking	266	32.2
	The middle	518	62.8
	The last one-third rankings	41	5.0
Screen time	<1 h	558	67.6
	1–2 h	216	26.2
	>2 h	51	6.2
Only child in the family	Yes	327	39.6
	No	498	60.4
Main caregiver	Parents	762	92.4
	Others	63	7.6
The habit of napping in the past month	Yes	347	42.1
	No	478	57.9

**Table 2:** PSQI total score and score details of each factor.

PSQI Scores	Minimum	Maximum	$\bar{x}$ ± *s*	Rank
Subjective sleep quality	0	3	0.88 ± 0.733	4
Sleep duration	0	3	0.96 ± 0.925	2
Sleep latency	0	3	0.15 ± 0.449	5
Sleep efficiency	0	3	0.10 ± 0.348	6
Sleep Disturbances	0	3	0.95 ± 0.541	3
Hypnotic medication use	0	3	0.02 ± 0.202	7
Daytime dysfunction	0	3	1.13 ± 0.898	1
Total scores	0	16	4.19 ± 2.545	

**Table 3:** Binary Logistic regression analysis of sleep quality of the primary students in grades 3–6.

	*β*	*SE*	Wald	*p*	*OR*	95% *CI*
Screen time	0.417	0.136	9.474	0.002	1.518	(1.164, 1.980)
Ranking status	−0.416	0.162	6.576	0.010	0.659	(0.480, 0.907)
Learning burnout	0.084	0.010	77.025	0.000	1.088	(1.067, 1.108)
Mental Health	1.542	0.445	12.012	0.001	4.672	(1.954, 11.173)
Constant	−5.026	0.570	77.836	0.000	0.007	

**Table 4:** Intermediary model test of mental health (*n* = 825).

Regression Equation (*n* = 825)		Fitting Index			Coefficient Significance	
Result Variable	Predictive Variable	R	R^2^	F(*df*)	*β*	*t*
Sleep quality		0.54	0.29	55.10		
	Age				0.328	1.724
	Grade				−0.164	−0.888
	Ranking status				−0.512	−3.443
	Screen time				0.318	2.411
	Whether they had the habit of napping in the past month				−0.477	−3.080
	Learning burnout				0.126	15.621
Mental health				61.64		
	Age				0.284	0.875
	Grade				−0.073	−0.232
	Ranking status				−0.728	−2.878
	Screen time				−0.035	−0.155
	Whether they had the habit of napping in the past month				−0.651	−2.472
	Learning burnout				0.241	17.587
Sleep quality				72.34		
	Age				0.268	1.507
	Grade				−0.148	−0.862
	Ranking status				−0.356	−2.556
	Screen time				0.326	2.649
	Whether they had the habit of napping in the past month				−0.337	−2.329
	Mental health				0.215	11.203
	Learning burnout				0.074	8.415

## Data Availability

Data cannot be shared publicly because of potentially identifying and sensitive participants information. Data are available from the Hunan Normal University of China (contact via powerestlulu@hunnu.edu.cn) for researchers who meet the criteria for access to confidential data.

## Supplementary Materials

The following supporting information can be downloaded at: https://www.mdpi.com/article/10.3390/healthcare10102076/s1, Table S1: PSQI scores 1; Table S2: PSQI scores 2; Table S3: PSQI scores 3; Table S4: ASBI total score and score details of each factor. Table S5: Describe statistics and correlation analysis results; Table S6: Breakdown of total effect, direct effect and intermediary effect.
