# Supplementary material for: The Effect of Learning Burnout on Sleep Quality in Primary School Students: The Mediating Role of Mental Health
Source: Healthcare (Basel). 2022 Oct 19;10(10):2076. doi: 10.3390/healthcare10102076 (PMC9602333; doi:10.3390/healthcare10102076)
Supplement: Supplementary file 1 [file healthcare-10-02076-s001.zip › healthcare-1964439-supplementary.pdf]

Table S1 PSQI scores 1

	N	**Minimum**	**Maximum**	$\bar{x}\pm s$
1. During the past month, when have you usually gone to bed at night?	825	8:00 PM	1:30 AM	9.7220±0.757
2. During the past month, how long(in minutes) has it usually take you to fall asleep each night?	825	1	99	19.19±16.067
3. During the past month, when have you usually gotten up in the morning?	825	5.00	8.00	6.8273±0.402
4. During the past month, how many hours of actual sleep did you get at night?	825	4.0	11.0	8.676±1.038

Table S2 PSQI scores 2 [*N*(%)]

	Not during the past month	Less than once a week	Once or twice a week	Three or more times a week
Q5a	447(54.2%)	131(15.9%)	116(14.1%)	131(15.9)
Q5b	423(51.3%)	174(21.1%)	130(15.8%)	98(11.9%)
Q5c	490(59.4%)	209(25.3%)	83(10.1%)	43(5.2%)
Q5d	724(87.8%)	58(7.0%)	26(3.2%)	17(2.1%)
Q5e	717(86.9%)	66(8.0%)	22(2.7%)	20(2.4%)
Q5f	607(73.6%)	112(13.6%)	69(8.4%)	37(4.5%)
Q5g	470(57%)	137(16.6%)	117(14.2%)	101(12.2%)
Q5h	458(55.5%)	181(21.9%)	101(12.2%)	85(10.3%)
Q5i	727(88.1%)	57(6.9%)	22(2.7%)	19(2.3%)
Q5j	664(80.5%)	67(8.1%)	53(6.4%)	41(5.0%)

Table S3 PSQI scores 3

	N	Percentage(%)
6. During the past month, how would you rate your sleep quality overall?		
Very good	255	30.9
Fairly good	442	53.6
Fairly bad	104	12.6
Very bad	24	2.9
7. During the past month, how often have you taken medicine(prescribed or over the counter)to help you sleep?		
Not during the past month	813	98.5
Less than once a week	8	1.0
Once or twice a week	2	0.2
Three or more times a week	2	0.2
8. During the past month, how often have you had trouble staying awake while driving, eating meals, o engaging in social activity?		
Not during the past month	329	39.9
Less than once a week	235	28.5
Once or twice a week	143	17.3

Three or more times a week	118	14.3
9. During the past month, how much of a problem has it been for you to keep up enough enthusiasm to get things done?		
No problem at all	312	39.9
Only a very slight problem	389	28.5
Only a very slight problem	88	17.3
A very big problem	36	14.3

Table S4 ASBI total score and score details of each factor

**Variables**	**Maximum**	**Maximum**	$\bar{x}\pm s$	**Ranking**
Mental health scores	0	24	4.46±4.404	_
Learning burnout scores				
Exhaustion	20	4	10.19±4.678	2
Learning cynicism	25	5	8.71±4.213	3
Reduced efficacy	31	7	17.33±4.726	1
Total	72	18	36.23±10.383	

Table S5 Describe statistics and correlation analysis results

	Learning burnout	Mental health	Sleep quality
Sleep quality	0.505**	0.551**	1
Mental health	0.542**	1	
Learning burnout	1		

Note: "**" means that at the level of 0.01 (two tails), the correlation is significant.

Table S6 Breakdown of total effect, direct effect and intermediary effect

	Effect	Boot *SE*	BootLLCI	BootULCI	Proportion in total effect (%)
Intermediary effect	0.052	0.007	0.039	0.065	41.27
Direct effect	0.074	0.009	0.056	0.092	58.73
Total effect	0.126	0.008	0.110	0.142	
